# First documented movement of a humpback whale between the Cape Verde Islands and West Greenland

**DOI:** 10.1002/ece3.11152

**Published:** 2024-03-15

**Authors:** Valerie Chosson, Virginie Wyss, Beatrice Jann, Frederick W. Wenzel, Guðjón Már Sigurðsson, Malene Simon, Rikke Guldborg Hansen, Lindsey S. Jones

**Affiliations:** ^1^ Marine and Freshwater Research Institute Hafnarfjörður Iceland; ^2^ Swiss Cetacean Society Lausanne Switzerland; ^3^ Swiss Whale Society Astano Switzerland; ^4^ Allied Whale College of the Atlantic Bar Harbor Maine USA; ^5^ NOAA National Marine Fisheries Service, Northeast Fisheries Science Center Woods Hole Massachusetts USA; ^6^ Greenland Climate Research Centre Greenland Institute of Natural Resources Nuuk Greenland; ^7^ Greenland Institute of Natural Resources København K Denmark

**Keywords:** breeding grounds, feeding grounds, migration, photo‐id, whaling – aboriginal

## Abstract

The endangered population of humpback whales (*Megaptera novaeangliae*) breeding and calving off the Cape Verde Islands (CVI) are known to migrate to feeding areas located along the eastern margin of the North Atlantic Ocean (Iceland, and Norway). Here, we report for the first time a confirmed migration of an individual humpback whale from CVI breeding ground to a western North Atlantic feeding ground of West Greenland. This individual humpback, which was photographed and identified off the coast of West Greenland in 2021, was previously documented in CVI 22 years before (1999). An annual subsistence hunt for humpbacks occurs in West Greenland and the resighting at this location with a humpback whale from CVI has strong implications for the conservation efforts of the small CVI population.

## Graphical abstract

The endangered population of humpback whales (*Megaptera novaeangliae*) breeding and calving off the Cape Verde Islands (CVI) are known to migrate to feeding areas located along the eastern margin of the North Atlantic Ocean (Iceland and Norway). Here, we report for the first time the confirmed migration of an individual humpback whale from CVI breeding ground to a western North Atlantic feeding ground, West Greenland. This individual humpback, which was photographed and identified off the coast of West Greenland in 2021, was previously documented in CVI 22 years before (1999). An annual subsistence hunt for humpbacks occurs in West Greenland, and the resighting at this location with a humpback whale from CVI has strong implications for the conservation efforts of the small CVI population.

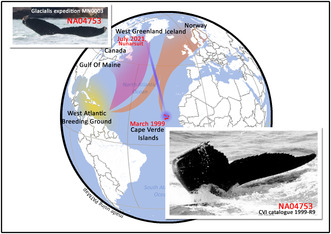

In the North Atlantic, humpback whales (*Megaptera novaeangliae*) use the breeding grounds of the West Indies (from Cuba to Venezuela; the Greater and Lesser Antilles) or the Cape Verde Islands (CVI), northwest Africa. The population of humpback whale breeding and calving off CVI includes fewer than 300 individuals (Punt et al., 2006). It is considered as discrete population of those from the Western margin of the Atlantic thus being one of the most endangered populations of humpback whale in the world (Bérubé et al., 2013) despite being considered at least concern by the International Union for Conservation of Nature (IUCN) (Cooke, 2018). The CVI population is likely a remnant of a historically larger breeding population in that area, which has not yet recovered from commercial whaling (Punt et al., 2006) and is listed as endangered under the US Endangered Species Act (ESA) (Reeves et al., 2001). The long‐distance migration patterns of North Atlantic humpback whales have been well‐studied using photographic‐identification (photo‐ID) techniques for nearly five decades. Humpback whale individuals are identified by their unique pattern on the ventral surface of their flukes (Schevill & Backus, 1960; Katona & Whitehead, 1981; Katona & Beard, 1990; Reeves et al., 2004; Smith et al., 1999). The North Atlantic Humpback Whale Catalogue (NAHWC) curated by Allied Whale at the College of the Atlantic (https://www.coa.edu/allied‐whale/) identifies over 11,000 individual humpback whales from the North Atlantic Ocean basin.

Five distinct feeding aggregations have been identified in different regions of the North Atlantic: Gulf of Maine, eastern Canada, West Greenland, Iceland, and northern Norway (Clapham & Mayo, 1987; Smith et al., 1999; Stevick et al., 2003). To date, humpback whales from the CVI breeding ground have only been confirmed on the high‐latitude feeding grounds of the eastern North Atlantic – Iceland and Norway (Figure 1) (Berrow et al., 2021; Jann et al., 2003; Wenzel et al., 2009, 2020). Humpback whales identified off the West Greenland feeding ground have previously been documented to migrate only to the breeding grounds of the West Indies (Jones et al., 2024; Perkins et al., 1984; Smith et al., 1999).

**FIGURE 1:**
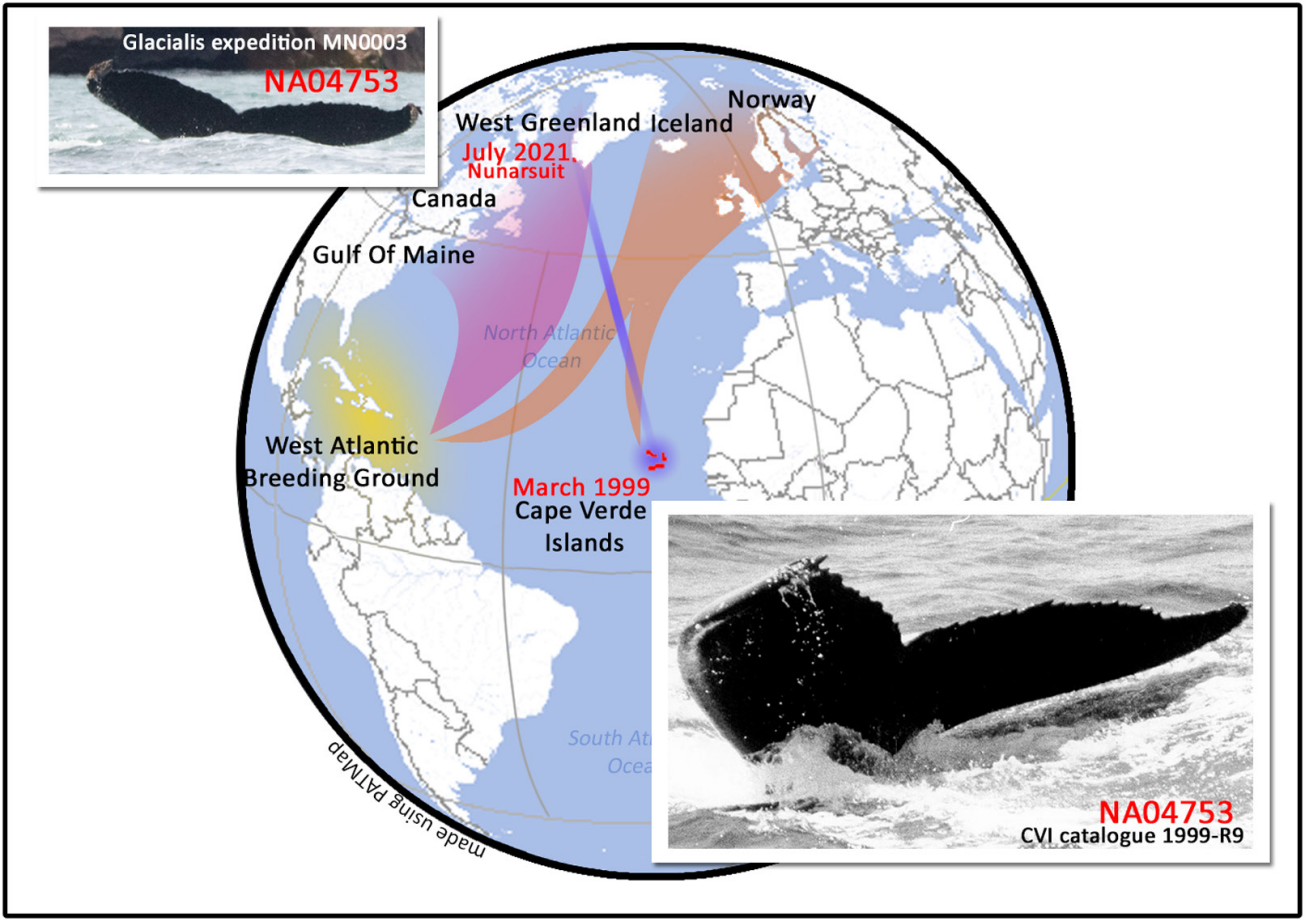
Locations where NA04936 was observed (Table 1). In March 1999 off the Cape Verde Islands and 22 years later, in July 2021 off Nunarsuit, Arsuk (West Greenland). The pink shade represents the breeding destination of humpback whale feeding in the Western feeding area (composed of the Gulf of Maine, Atlantic Canada, and West Greenland feeding grounds). The orange shaded areas represent the breeding destinations of humpback whale feeding at the Eastern feeding grounds. The yellow shaded region represents the breeding grounds in the West Indies. The top left corner shows a photograph of NA04936 taken on the 4th of July 2021 (©Glacialis expedition), and the bottom right corner, a photograph of NA04936 taken on the 24th of March 1999 of the Cape Verde Islands (©Beatrice Jann).

**TABLE 1:** Sightings (date and location) and original identification of NA04936.

Original catalogue	Individual local ID	Sighting		
		Date (yyyy‐mm‐dd)	Latitude	Longitude
Cape Verde	1999‐R9	1999‐03‐24	NA	NA
Glacialis	MN0003	2021‐07‐04	N60°40	W47°53

Abbreviation: NA, non‐available.

In West Greenland, Baffin Island Current who carry cold waters from the Arctic Ocean mixes with West Greenland current and the warmer and saltier North Atlantic waters. This creates a highly productive zone in which a large variety of humpback whales prey items such as capelin (*Mallotus villosus*), sandeels (*Ammodytes marinus*), and euphausids thrive (Laidre et al., 2015; Larsen & Hammond, 2004). Since 1984, the abundance of humpback whales summering at the West Greenland feeding ground has been estimated by regular aerial line transect survey and has steadily increased by 9.4% per year up to 2007 (Laidre & Heide‐Jørgensen, 2012). This regular increase of the West Greenland feeding aggregation is of the same magnitude as the one observed in other North Atlantic feeding grounds (Heide‐Jørgensen et al., 2012).

The Greenland Institute of Natural Resources (GINR) curates a catalogue of humpback whale individuals for the West and East Greenland feeding aggregations. This represents a collection of 466 individuals in West Greenland. To date, the individuals of this catalogue have been compared to the NAHWC and catalogued.

In the summer of 2021, the Expedition Glacialis documented a total of 44 humpback whale sightings off West Greenland during a voyage from the Azores archipelago (N38°32; W28°37) to the Baffin Sea and Cape Thorvaldsen (N60°40; W47°53) (Figure 2). Out of these 44 observations, a total of 28 individuals (Table 2) were identified based on photographs of their flukes and compared to the NAHWC using a combination of automated image recognition and manual visual comparison methods (Cheeseman et al., 2022; Katona & Whitehead, 1981). On the 4th of July 2021, off Nunarsuit (near Arsuk), Greenland (N60°40; W47°53), four humpback whale individuals were encountered, and photo‐ID images were obtained. One of these individuals was identified as NA04936 in the NAHWC (Figure 2). Its only previous documented sighting occurred in 1999 off the CVI coast. No other sightings of this individual have been documented to date.

**FIGURE 2:**
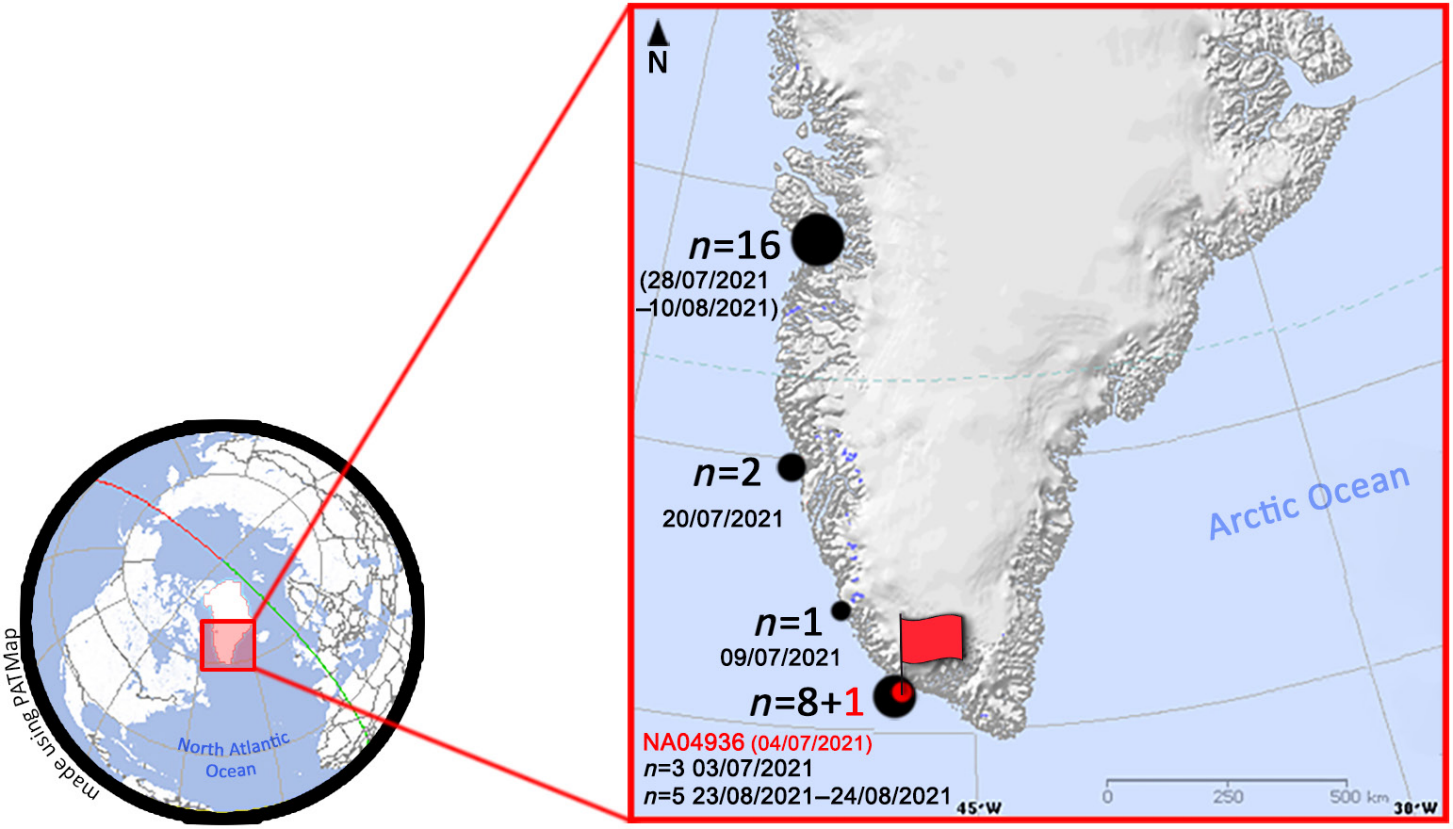
Location of the 28 sightings of humpback whale during the Glacialis expedition off the West Greenland coast. Dates indicate the day or the period of the sightings and *n*, the number of humpback whales observed and photographed. The red flag marks the NA04936 sighting location on 4th of July 2021 (Table 2).

**TABLE 2:** Records of the 28 sightings of humpback whale individual photo‐identified off the coast of West Greenland during the Expedition Glacialis (Figure 2).

	Glacialis ID	Sighting		
		Date (yyyy‐mm‐dd)	Latitude	Longitude
	GLACIALISMN0001	2021‐07‐03	N60°40	W47°53
	GLACIALISMN0025	2021‐07‐03	N60°40	W47°53
	GLACIALISMN0002	2021‐07‐03	N60°40	W47°53
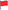	GLACIALISMN0003 ^a^	2021‐07‐04	N60°40	W47°53
	GLACIALISMN0024	2021‐07‐09	N62°04	W47°16
	GLACIALISMN0004	2021‐07‐20	N64°43	W52°23
	GLACIALISMN0026	2021‐07‐20	N64°51	W52°11
	GLACIALISMN0005	2021‐07‐28	N68°6	W53°18
	GLACIALISMN0006	2021‐07‐29	N68°6	W53°18
	GLACIALISMN0007	2021‐08‐04	N68°5	W51°36
	GLACIALISMN0008	2021‐08‐05	N69°07	W51°06
	GLACIALISMN0011	2021‐08‐05	N69°07	W51°06
	GLACIALISMN0009	2021‐08‐05	N69°10	W51°07
	GLACIALISMN0010	2021‐08‐05	N69°10	W51°07
	GLACIALISMN0008	2021‐08‐07	N69°10	W51°07
	GLACIALISMN0011	2021‐08‐07	N69°10	W51°07
	GLACIALISMN0012	2021‐08‐07	N69°10	W51°07
	GLACIALISMN0013	2021‐08‐07	N69°10	W51°07
	GLACIALISMN0014	2021‐08‐07	N69°10	W51°14
	GLACIALISMN0015	2021‐08‐07	N69°10	W51°13
	GLACIALISMN0016	2021‐08‐08	N69°23	W50°6
	GLACIALISMN0017	2021‐08‐10	N69°11	W51°08
	GLACIALISMN0018	2021‐08‐10	N69°10	W51°07
	GLACIALISMN0019	2021‐08‐23	N60°40	W47°55
	GLACIALISMN0020	2021‐08‐23	N60°40	W47°55
	GLACIALISMN0021	2021‐08‐24	N60°40	W47°52
	GLACIALISMN0022	2021‐08‐24	N60°41	W47°46
	GLACIALISMN0023	2021‐08‐24	N60°42	W47°35

*Note*: The red flag marks the observation date and coordinates of GLACIALISMN0003, known as NA04936 in the NAHWC and 1999‐R9 in the Cape Verde Catalogue of 1999 (Table 1).

^a^ NA04936.

Between the years 1990 and 2001, when photo‐ID effort first began in CVI, only 42 unique humpback whales were documented and identified in CVI waters with ventral fluke photographs (Jann et al., 2003). With a total of 22 individuals registered in 1999 and only 3 prior to that year, NA04936 is among one of the first humpback whale individuals registered in the CVI catalogue (Jann et al., 2003). This match spans a period of 22 years. It is the first confirmed movement of an individual from the endangered humpback whale population of CVI and the West Greenland feeding ground. This feeding ground is an important foraging area for humpback whales while also a subsistence hunting ground under the International Whaling Commission (IWC) regulations (IWC, 2018), with a current annual quota of 10 humpback whales. With a recent observed change in humpback whale distribution around Greenland (Hansen et al., 2018; Heide‐Jørgensen & Laidre, 2015), a lower quantity of humpback whales aggregate at the West Greenland feeding ground as some whales move to the East Greenland feeding area. That increases the probability of an individual whale to be hunted, hence raising conservation concern on the small and endangered CVI humpback whale population.

As 22 years passed between the two sightings, the relatively strong feeding ground fidelity known in this species (Reeves et al., 2001) and especially in this region (Larsen & Hammond, 2004) raises questions about long‐distance migration of this individual. Continuous and regular efforts in West Greenland to collect sightings, photo‐ID, and genetic data on humpback whales aggregating on the feeding ground would shed a better light on their ocean‐basin‐wide movement patterns. Thus, it would help to better understand the dynamic of humpback whale migration patterns in the North Atlantic. It will also unravel the importance of the presented match for the conservation and management of the endangered CVI population. The recovery of the small population using the CVI breeding and calving grounds needs to be continually monitored and assessed considering possible changing movement patterns and as the population continues to face anthropogenic threats and environmental changes throughout their range.

## AUTHOR CONTRIBUTIONS

**Valerie Chosson:** Conceptualization (lead); data curation (equal); formal analysis (equal); investigation (equal); project administration (equal); resources (equal); visualization (equal); writing – original draft (equal); writing – review and editing (equal). **Virginie Wyss:** Funding acquisition (equal); investigation (equal); resources (equal); writing – review and editing (equal). **Beatrice Jann:** Formal analysis (equal); funding acquisition (equal); resources (equal); writing – review and editing (equal). **Frederick W. Wenzel:** Data curation (equal); formal analysis (equal); writing – review and editing (equal). **Guðjón Már Sigurðsson:** Funding acquisition (equal); resources (equal); writing – review and editing (equal). **Malene Simon:** Formal analysis (equal); resources (equal); writing – review and editing (equal). **Rikke Guldborg Hansen:** Formal analysis (equal); resources (equal); writing – review and editing (equal). **Lindsey S. Jones:** Conceptualization (supporting); data curation (lead); formal analysis (equal); funding acquisition (equal); investigation (equal); resources (equal); writing – review and editing (equal).

## CONFLICT OF INTEREST STATEMENT

The authors declare no competing interests.

## Data Availability

Open access metadata in Center for Open Science . Data and materials are also available upon request to the first author or the North Atlantic Humpback Whale Catalogue (nahwc@coa.edu; Allied whale). Expedition Glacialis Data are also available upon request to the first author (valerie.chosson@hafogvatn.is) and at Glacialis expedition .
